# Histological characteristics of dental pulp in teeth with periodontal problems and healthy teeth

**DOI:** 10.34172/japid.2023.005

**Published:** 2022-12-28

**Authors:** Farzane Vaziri, Ahmad Haerian, Laleh Maleki, Samane Abbasi

**Affiliations:** ^1^Department of Periodontics, School of Dentistry, Shahid Sadoughi University of Medical Science, Yazd, Iran; ^2^Department of Oromaxillofacial Pathology, School of Dentistry, Isfahan University of Medical Sciences, Isfahan, Iran; ^3^Department of Periodontics, School of Dentistry, Hormozgan University of Medical Science, Bandar Abbas, Iran

**Keywords:** Chronic periodontitis, Dental pulp, Histopathology

## Abstract

**Background.:**

Chronic periodontitis is the most common type of periodontitis, which is associated with calculus and plaque accumulation. Several studies have indicated that uncured periodontitis can affect the dental pulp. However, this relationship is controversial. This study aimed to compare histopathological results obtained from the dental pulp in teeth with periodontal problems and healthy teeth.

**Methods.:**

In this study, 23 teeth with periodontal problems and 23 healthy teeth were extracted. After cutting off 2 mm from the root apex, the teeth were sectioned into apical, middle, and coronal thirds. Then, the specimens were evaluated in both groups based on histopathological features.

**Results.:**

According to the evaluations, in the middle third, the amount of inflammation was only statistically significant in the group that consisted of teeth with periodontal problems (*P*=0.014). There was no significant difference in fibrosis and blood vessel diameter between the two groups. Necrosis in the middle (*P*=0.002) and coronal thirds (*P*=0.004) of teeth with periodontal problems was more than the healthy teeth. The lack of odontoblastic integrity in all the sections of teeth with periodontal problems was more than that in healthy teeth (*P*=0.0001).

**Conclusion.:**

Inflammation of the periodontium in chronic periodontitis can lead to histological changes in the pulp, including an increase in inflammation, necrosis, and loss of odontoblastic integrity.

## Introduction

 Periodontitis is an inflammatory disease of supporting periodontal structures which results in the progressive destruction of the periodontal ligament and alveolar bone.^1^ Chronic periodontitis is the most common form of periodontitis with high prevalence.^2^ From an anatomic viewpoint, the periodontium is related to dental pulp through the apical foramen and accessory canals. These relationships facilitate the transmission of pathogenic agents between the pulp and periodontium. Therefore, the pathologic changes in the pulp or periodontium can affect the other.^3-5^ Some studies have confirmed the relationship between the pulp and periodontium through the apical foramen and accessory canals.^6-8^ According to Adriaens, 59% of teeth suffering from periodontitis had the same bacteria in the pulp and periodontium.^6^ Several previous studies demonstrated that untreated periodontal disease could affect the dental pulp and cause endodontic lesions.^3,9^ Pulpal changes following periodontal inflammation not only affect the health of the pulp but also can affect the outcomes of root canal therapy.^10^ Some studies have shown that periodontitis causes a reduction in the pulp volume by approximately 20%.^11^ The relationships between periodontal infection and pulp pathosis are still a matter of debate, and many studies have shown different results.^3,12,13^ Considering the differences in the results and limited studies on the effects of periodontal disease on dental pulp, especially on human teeth, this study compared the histological characteristic of dental pulp in teeth with periodontal problems and healthy teeth.

## Methods

 This laboratory study included 23 anterior teeth with periodontal problems and 23 healthy teeth. This study was approved by the Ethics Committee of Yazd Shahid Sadoughi Dental School. Inclusion criteria for this study were teeth with an attachment loss of > 6 mm, a crown-to-root ratio of > 1:1, teeth with a hopeless prognosis, and teeth with grades II and III mobility. In the control group, teeth with healthy periodontium were extracted for reasons like full extraction for a complete denture or orthodontic reasons in adults. In both groups, the teeth were intact, without caries or history of trauma, restoration, bruxism, and periapical lesions on radiographs. All the patients were systemically healthy and nonsmokers. Before extraction, each tooth underwent a careful periodontal examination. Chronic periodontitis was diagnosed based on the American Academy of Periodontology criteria.^14^ Examinations included measuring the attachment level by Williams’ probe with 1-mm accuracy (distance from the CEJ to the pocket depth) and tooth mobility by two metallic devices. After local anesthesia, the teeth were atraumatically extracted. Immediately after extraction, the apical 2‒3 mm of the root was cut using a fissure diamond bur in a high-speed handpiece under cool water irrigation, followed by immersion in 10% formalin solution (Dr. Mojallali Industrial Chemical Co., Iran) for seven days. Cutting the root end provided clear access to fix the pulp.^10^ Then the teeth were decalcified in 5% HNO3 (Mojallali Industrial Chemical Co., Iran) for 10 days. The specimens were embedded in paraffin and sectioned with a microtome into the apical, middle, and coronal thirds, measuring 5 μm in thickness. Then the specimens were mounted and stained with hematoxylin and eosin (H&E). After histological processing, the sections were examined by an experienced pathologist under a light microscope (Model ECLIPSE E200, NIKON, Japan). The relative amount of inflammation, fibrosis, edema, condition of the pulpal vessels, and odontoblastic integrity were examined. The relative amount of inflammation was graded as follows: no inflammation (0‒2 inflammatory cells), mild inflammation (2‒5 inflammatory cells), moderate inflammation (5‒10 inflammatory cells), or severe inflammation ( > 10 inflammatory cells).^13^ The relative amount of fibrosis was graded as follows: mild fibrosis (3‒10 fibroblasts), moderate fibrosis (11‒30 fibroblasts), or severe fibrosis ( ≥ 31 fibroblasts).^13^ Necrotic specimens were categorized as partial necrosis, complete necrosis, or no necrosis. The vessels were categorized into three groups (normal, atrophic, or dilated) according to their size.^3^ After collecting all the data, the chi-square test was used to analyze the data.

## Results

**Figure 1:**
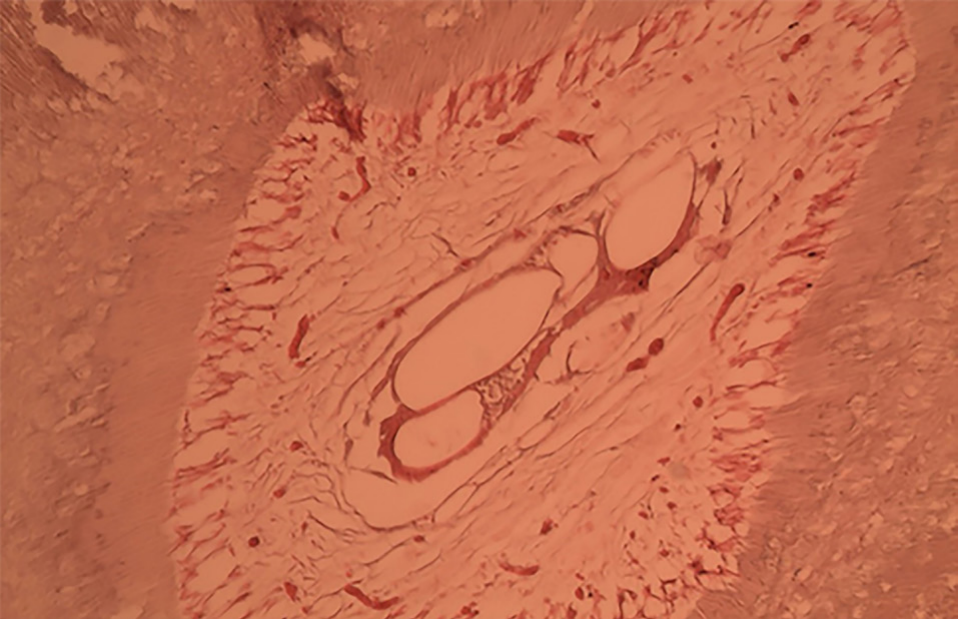
A sample of the histological image of inflammatory cells and dilated vessels in the case group

 This laboratory case‒control study evaluated the effects of chronic periodontitis on histological parameters of the pulp tissue. The percentages of inflammatory cells in each section are shown in Table 1. Figure 1 shows the histological features of inflammatory cells in teeth with periodontal problems. According to the chi-squared test, the difference between the amount of inflammation in apical sections was not statistically significant (*P* = 0.063). This difference was statistically significant in the middle section (*P* = 0.014), but in the coronal section, the difference was not statistically significant (*P* = 0.068).

**Table 1:** The percentages of inflammation in both groups with healthy teeth and teeth with periodontal problems in all three sections

**Section**	**Degree of inflammation**	**Healthy group**	**Periodontitis group**	***P *** **value**
Apical	No	73.9%	52.2%	0.063
	Mild	26.1%	26.1%	
	Moderate	17.4%	0%	
	Severe	4.3%	0%	
Middle	No	82.6%	47.8%	0.014
	Mild	13%	30.4%	
	Moderate	0%	13%	
	Severe	4.3%	8.7%	
Coronal	No	78.3%	52.2%	0.068
	Mild	13%	30.4%	
	Moderate	8.7%	8.7%	
	Severe	0%	8.7%	

 Mild fibrosis of the pulp was a common finding in the case and control group in all the coronal, middle, and apical sections (87% in the apical section and 78.3% in the middle and coronal sections). Most of the specimens in the case group also demonstrated mild fibrosis. According to the chi-squared test, there was no significant difference in any sections between the two groups (*P* > 0.05). Blood vessel diameters of most teeth were normal in both groups. Atrophic vessels in teeth with periodontal problems were shown in 21.7% of the apical and coronal sections and 39.1% of the middle sections. These changes in the control group included 4.3% in the apical section and 8.7% in the coronal section. 100% of the controls had normal blood vessel diameters in the middle section. Dilated vessels in the case group were seen in 26.1% of the apical sections and 21.7% of the middle and coronal sections. In the control group, 4.3% of the apical and coronal sections exhibited dilated vessels. According to the chi-squared test, there was no significant difference in any sections between the two groups (*P* = 0.071). There was no pulp necrosis in 95.7% of healthy teeth in the apical section, while 78.3% of the specimens were without necrosis in the periodontal problem group. The chi-squared test showed no significant difference between the two groups in this section (*P* = 0.083). All the teeth in the healthy group in the middle third and 95.7% of the samples in the coronal third were without necrosis. However, in the case group, 65.2% of the specimen in the middle and 79.3% of the samples in the coronal section showed no necrosis. According to the results, there was a significant difference between the two groups in the middle and coronal sections (*P* = 0.002, *P* = 0.004). The differences in odontoblastic layer integrity in both the case and control groups in all three sections were significant (Table 2) (*P* = 0.001).

**Table 2:** Odontoblastic layer integrity in healthy teeth and teeth with periodontal problems in all the three sections

**Section**	**Odontoblastic layer integrity**	**Healthy group**	**Periodontitis group**	***P*** **value** ^a^
Apical	No	13%	65.2%	0.0001
	Yes	87%	34.8%	
Middle	No	13%	60.9%	0.0001
	Yes	87%	39.1%	
Coronal	No	17.4%	69.6%	0.0001
	Yes	82.6%	30.4%	

^a^Chi-square test.

## Discussion

 The relationship between periodontal disease and pulp damage has always been discussed. In this study, we evaluated the possible effects of moderate-to-severe chronic periodontitis on different aspects of histological pulp structure. Considering the absence of a control group in most previous studies and the lack of comparison of the results with the results obtained from samples without periodontitis, we can claim that this study is one of the pioneers in this field that compared the results between case and control groups. The results of the present study showed that, in the case group, pulp necrosis, not the integrity of the odontoblastic layer and inflammation were more than that in the control group. There were no significant differences between the two groups in other parameters. However, there was some degree of fibrosis, dilated pulpal vessels, and calcification in some sections of both groups. Huanga and Chena^15^ reported pulpal tissue calcifications in 122 (62%) out of 197 specimens. Indeed, 56% of teeth with calcification were molars, while our study samples were single-rooted teeth.

 Fatemi et al^13^ evaluated the effect of chronic periodontitis on dental pulp. Inclusion criteria in their study were bone loss > 6 mm, a crown-root ratio of > 1:1, mobility grades of II and III, and the absence of gingival recession; however, the absence of gingival recession was not considered in the present study. Tooth preparation, like cutting 2‒3 mm of the root end for immersion in formalin solution to fix the pulp tissue, was similar in both studies. Fatemi et al did not mention which section of the root was prepared, while in the present study, three coronal, middle, and apical sections of the root were prepared and compared separately. In the study by Fatemi et al, inflammation was a common finding, and only 6.3% of teeth were free of inflammation; however, in our study in the case group, 50% of teeth showed no inflammation in 37 sections. The differences might be attributed to the different inclusion criteria of the samples in the two studies. A history of periodontal treatment or denuded root surfaces can increase the chance of pulpal inflammation.^16^ The presence of accessory canals and the relationship between the pulp and periodontium depend on the thickness of the cementum, the presence of exposed dentin, the denuded root surface, prior periodontal treatment, and frequent root planing. These factors can affect inflammatory changes, which were not considered in both studies. Also, the presence of inflammation in some teeth is attributed to artifacts, tissue folding, and uneven odontoblastic layer, which was misdiagnosed as lymphocytes and inflammatory tissues in prepared sections.^17^ Caraivan et al^18^ reported increased chronic inflammatory cells in the dental pulp in teeth with chronic severe periodontitis. In Caraivan and colleagues’ study, smokers and diabetic patients participated, which were excluded from our study. Considering the effects of smoking and diabetes on vessels and immune function, we can consider them confounding factors in their study.^18^ Heidari et al^19^ investigated stereological indices of the dental pulp in patients with advanced periodontitis compared with healthy individuals. This study was similar to our study regarding the control group. In Haidari and colleagues’ study, the specimens were sectioned longitudinally and stained with Masson’s trichrome, which was different from ours. According to their results, inflammation in the case and control groups was not significantly different. In the present study, the specimens were sectioned in a transverse direction into three coronal, middle, and apical segments; there was a significant difference in the middle section. Sletzetr’s hypothesis indicated that retrograde pulpitis interference of periodontal lesions with the nutritional supply of the tooth apex leads to atrophic and degenerative changes in the pulps of the affected tooth. In severe periodontitis with tooth mobility and bone loss, which leads to the periodontal lesion in the dental apex, this hypothesis could justify these inflammatory, degenerative, and necrotic changes.^17^

 Mild fibrosis was prevalent in Fatemi and colleagues’ study.^13^ In our study, mild fibrosis was seen in frequent specimens in both the case and control groups. Since there was no significant difference between the two groups, we cannot attribute this finding to periodontitis. Age, trauma from occlusion, and so on could cause fibrosis in both groups.^20^ Caraivan and colleagues’^18^ study also showed condensing collagen fibers and a large number of fibroblasts in chronic periodontitis^;^ however, in Fatemi and colleagues’ study^13^ and the present study, the number of fibroblasts was considered, not the condensation of collagen fibers. Arterial changes in Fatemi and colleagues’ study were dilated and atrophic arteries. Our study showed the same alterations in both the case and control groups, and the differences were not significant. In Caraivan and colleagues’ study, arterial changes included vessel remodeling changes like fewer blood vessels and atherosclerotic changes in moderate chronic periodontitis patients.^18^ As mentioned before, smokers and diabetic patients participated in the study, which could justify these changes. Necrotic changes were seen in Fatemi and colleagues’^13^ study in several sections, in which partial necrosis was more prevalent than complete necrosis. They did not find any necrosis in 35.4% of sections. In the present study, partial necrosis was seen in all the sections, with no complete necrosis in any section. In an animal study, Nemec et al^21^ showed that the pulps of dog teeth affected by periodontitis were frequently inflamed and necrotic, which might be attributed to advanced periodontitis affecting these teeth or a mechanical effect related to tooth mobility, consistent with our study. Necrosis and degenerative changes might be attributed to the invasion of the periodontal lesion by arterial innervations of the tooth apex. However, degenerative changes reported in many past studies were due to insufficient fixation and no complete penetration of formalin into the dental pulp tissue.^17,22^ Necrotic and inflammatory changes, via pumping plaque metabolites, can be attributed to tooth mobility due to periodontitis.^23^ Although many studies have reported the effect of periodontitis and inflammation of supporting periodontal tissues on the dental pulp, some studies have not shown this relationship.^23-26^ It is still unknown whether the observed changes are limited to patients’ teeth suffering from periodontitis or whether other teeth of the same patient are also affected. It is very difficult to design a study where the members of the case and control groups are both from the same patient in human studies; therefore, the answer to this question will be limited to animal studies.

## Conclusion

 In conclusion, our results indicate that moderate-to-severe chronic periodontitis can affect some histological dental pulp parameters, such as a lack of integrity of the odontoblastic layer and necrosis and, to a lesser degree, dental pulp inflammation.

## Acknowledgments

 This study was financially supported by Yazd Shahid Sadoughi University of Medical Sciences, Vice Chancellor for Research and Technology.

## Availability of Data

 The data from the reported study are available upon request from the corresponding author.

## Competing Interests

 The authors declare no competing interests.

## Ethical Approval

 The present study has the code of ethics IR.SSU.REC.1394.98 of Shahid Sadoughi University of Medical Science, yazd, Iran.

## Funding

 None.
